# The Deadly(?) Cigarette

**Published:** 1898-06

**Authors:** 


### THE DEADLY(?) CIGARETTE.

The evils of the little cigarette have been told over and over again so often, and have been pictured so vividly under terrifying head-lines in the lay press, that the public almost believes it to be capable of producing all diseases and instigating all crimes. It is thought to contain a large host of captivating and death-dealing poisons and to promote all inordinate and sinful affections. We believe that these ideas have existed and do now exist principally, if not entirely, among the uninformed, and we are pleased to see that there are not wanting capable men who are willing, for truth's sake, to say an honest word in the cigarette's defense. The chief charges against the cigarette have been that they contain harmful ingredients (other than tobacco) and that excessive indulgence causes insanity and other mental diseases.

In the December number of the *Medico-Legal Journal* (New York) appeared a paper entitled "A Brief for the Cigarette," by William H. Garrison, Esq., which combated in a conclusive manner the "prevalent popular prejudice against the cigarette." He showed (1) that the domestic cigarette is composed of paper and tobacco, and of nothing else; and (2) that there is no good reason to believe that the cigarette ever caused a case of insanity. The author quoted a number of reports from expert chemists as to the quality of the tobacco and paper, and these unanimously testified to the "entire absence of foreign matter, deleterious or otherwise." (To the same effect we remember to have read an interesting paper some months ago by Dr. A. R. Ledoux, a distinguished chemist of New Jersey.)

As the paper of Mr. Garrison was somewhat incomplete touching the alleged relations of the cigarette to insanity and allied disorders, the editor of the *Medico-Legal Journal* collected opinions from asylum superintendents and other medical men having large experience with the insane; the greater part of these opinions are published in the March number of the above named journal. In the letters of these doctors (who must be accepted as best qualified to speak on this matter) we find such thoughts as these:

"The popular prejudice against the cigarette, I am sure, is false."

"I have long entertained the conviction that tobacco in various forms has been unjustly charged with many undeserved malign influences. During the past twenty years, the term of my devotion to the care of the insane, I have known over 4,000 insane patients, and so far as I can at present remember, in no single case was tobacco in any form answerable, directly or indirectly, for the mental disease."

"I have had an experience of fourteen years in the care of the insane, and several thousand cases of insanity have passed under my observation in that time. I have never yet seen a case of insanity that I believed was caused by cigarette smoking."

"There is no foundation of facts for the popular prejudice against the cigarette. From personal analysis and examination of the constituents of the cigarette, as well as a knowledge of the results experienced by expert chemists throughout the country, I am satisfied that only tobacco is used for filling, and the wrappers are made of the finest quality of paper possible to manufacture. After an observation [extending over twenty years] of many thousand cases of insanity, in persons of all ages, I can recall none caused by the use of the cigarette, or, I might add, tobacco in any form."

